# Lady Dufferin's Fund

**Published:** 1888-04-07

**Authors:** 


### Lady Dufferin's Fund.

One of the most interesting contributions that has recently been made to the literature of hospitals is the report of the National Association for Supplying Female Medical Aid to the Women of India, better known to the English public as Lady Dufferin's Fund. It is a record of continued progress, the more noteworthy that it is made in spite of many difficulties. The prejudice against new methods of treatment and European practitioners, and the jealousy of native doctors whose pretensions are now discredited, are no mean obstacles; and that they have been overcome in so large a degree proves most conclusively the patience, energy, and efficiency of all connected with the association, from the noble lady who founded it, and the native princes who have aided her with both money and influence, to the least important subordinate in the remotest branch association. The support which the native rulers and officials have given to the association is one of its most gratifying features, as proving both that they appreciate to the full the advantages of European medical treatment, and that the association is keeping closely to its promise of being unsectarian, and purely medical in its work. The report says distinctly that the National Association "cannot employ missionaries, nor can it provide hospital accommodation in which it is intended to combine medical treatment with religious teaching." That comparative failure is the result of an attempt to combine medical treatment with proselytism is shown by the complaint of Miss Beatty, M.D., of the Canadian Mission, Indore, that few except low-caste women attend her City Dispensary. However great the respect in which we hold medical missionaries, it is evident that any association which pretends to be national must conscientiously refrain from tampering with religious convictions, or, indeed, with anything outside its own work. Lady Dufferin and her helpers have great reason to be proud of the testimony to the strictly unsectarian character of their efforts which is borne by the fact that the Pundit Sailajamund Ojha, High Priest of the Hindu temples at Baidyanath, has offered a scholarship of 150 rupees per annum, tenable for three years, to be awarded to a Hindu female student at the Calcutta Medical College, who shall belong to one of the five high castes. Encouraged and supported by persons of distinction belonging to the Christian, Mohammedan, and Hindu religions, the association cannot fail in time to get rid of the untrained native doctors and midwives, of whose methods of practice a slight but sufficient specimen is given by Surgeon-Major D. F. Keegan, of Indore, on page 85 of the report.

That this desirable result will need both time and patience is undeniable. "You must creep before you gang," says the North-country proverb; and in some of the remoter districts the association is still in the creeping stage. Fully qualified medical women—European or native—are not employed, because there are not funds at command to pay them; and in such cases "hospital assistants," whose training leaves something to be desired, are all that can be provided. The association has had on this score to suffer some severe criticisms from people who have not fully understood the circumstances. Dr. Sophia Jex-Blake, a lady whose remarks on any subject connected with medical women must be received with respect, writing in the *Nineteenth Century* for November, expresses the opinion that the association "commits a very serious error in accepting partially-qualified women, and especially the lower class of medical practitioners educated at the Indian colleges with a much-restricted curriculum, and in placing them practically on an equal footing with the graduates of these same colleges or of European schools." This statement shows inaccuracy as well as misunderstanding. With regard to the employment of these imperfectly qualified women, Lady Dufferin truly says that "the choice does not lie between the most highly-educated lady doctor and the hospital assistant, but between the hospital assistant and no medical attendance whatever. The day will never come when highly-educated women of any race will settle down in country villages and accept four annas for a fee. We (the association) want women who will do this; and who, if unable to undertake those serious cases for the treatment of which rich people rush up to London or Calcutta, are yet fully competent to deal with the hundreds of minor maladies which are so much more common and oftentimes so distressing."

Nor is it intended that these hospital assistants shall work independently. They are to act, so far as possible, under competent supervision; and in districts where there is no qualified lady doctor, a female hospital assistant can do much, not only by her own attendance, but by intelligently reporting the symptoms of a case to a male practitioner and thus bringing to it the advantages of his skill and experience. Nor must it be forgotten that even a very little knowledge of medicine is better than the worse than ignorance possessed by the native "dhaies" or midwives, who were the only doctors the women of India have had till lately. As the secretary of one of the branches says: "If the dhaies could be trained to even that degree of ignorant efficiency possessed by an English peasant-woman, who helps her neighbour, a great deal would be gained."

The training of midwives is an important part of the work of the association, and one that will perhaps do more to win the suffrages of the people of India than any other department of medical training. But midwives alone are not sufficient, for in some cases the trained doctor is not called in till the "dhai" has failed in her task. In Surgeon-Major Goldsmith's report from Central India, he states that though Mrs. Talbot, the trained midwife at Rewah, has won the respect of the people, and has, though with some difficulty, formed a class for dhaies, there has been considerable disappointment that she is not a practitioner qualified to treat general diseases. "I feel sure," says Dr. Goldsmith, "that a well-qualified lady practitioner would soon obtain a large practice;" and he thinks that such a one would, if she first proved her skill in difficult cases of disease, be entrusted with obstetric practice.

The formation of hospitals is the department of the work of the association in which most difficulty is experienced. As yet patients have to be persuaded to come to the hospitals; but it may reasonably be supposed that those who have experienced the care and skill to be found there will induce others to profit by them, and at the same time to afford the students needed opportunities of clinical instruction.

Difficulties must, indeed, be looked for in the prosecution of such a scheme as this; but if they are encountered in the same honest and practical spirit which has hitherto been shown, we cannot doubt—the remarkable progress already made in three years sufficiently proves—that this Association, which has already won so high a place in the respect and esteem of both East and West, will go on increasing its beneficent influence, and will save from danger, pain, and death the women of our Indian Empire.

